# Effects of dexamethasone on TNF-alpha-induced release of cytokines from purified human blood eosinophils

**DOI:** 10.1186/1476-7961-3-5

**Published:** 2005-04-27

**Authors:** Iain Uings, Ilaria Puxeddu, Vladislav Temkin, Susan J Smith, Dilniya Fattah, Keith P Ray, Francesca Levi-Schaffer

**Affiliations:** 1 Cell Biology Unit, Glaxo Wellcome SKB, Gunnels Wood Stevenage, Herts, SG1 2NY, UK; 2 Department of Pharmacology, School of Pharmacy, The Hebrew University of Jerusalem, Jerusalem, Israel

**Keywords:** TNF-α, eosinophils, dexamethasone, allergic inflammation, cytokine

## Abstract

**Background:**

TNF-alpha is an important mediator in allergy also for its effects on eosinophils.

**Methods:**

The effect of dexamethasone on TNF-alpha induced eosinophils survival, degranulation (ECP), cytokines release (IL-8, GM-CSF) and adhesion to VCAM-1, ICAM-1 and IgG coated wells (EPO release) were evaluated.

**Results:**

The drug inhibited IL-8 and GM-CSF production, but not viability, degranulation or adhesion in human peripheral blood eosinophils.

**Conclusion:**

These results indicate that part of the activity of glucocorticosteroids on eosinophils may be mediated by their ability to inhibit cytokine secretion that in turn is important for the perpetuation of the allergic inflammation.

## Background

Eosinophils are bone marrow-derived granulocytes that play a crucial role in allergic inflammation. TNF-α is a pro-inflammatory cytokine synthesized by many inflammatory and structural cells. We previously demonstrated that mast cell-derived TNF-α induced eosinophil survival by autocrine production of GM-CSF [1]. TNF-α is also involved in eosinophil adhesion to endothelial cells and induces eosinophil activation, degranulation, and cytokines production. Glucocorticosteroids (GCS), the main anti-inflammatory drugs in allergic diseases, have been demonstrated to decrease circulating and tissue eosinophils. *In vitro *dexamethasone can inhibit eosinophil survival [2], expression of adhesion molecules [3], and cytokines production [4]. However, the effect of GCS on TNF-α induced eosinophil activation has only been partially investigated. The present study evaluated the effect of dexamethasone on TNF-α induced eosinophil degranulation, cytokines release and adhesion to VCAM-1, ICAM-1 and IgG.

## Materials and Methods

### Cells culture

Eosinophils were purified (>95%) from the peripheral blood of healthy non-atopic volunteers as previously described [5]. Freshly isolated eosinophils (viability >98%) were cultured in 96 well flat bottom tissue culture plates (Costar, High Wycombe, UK) (1.5 × 10^5^/200 μl/well) in RPMI-1640 supplemented with 10% heat inactivated foetal calf serum (FCS) containing 2 mM L-glutamine, 100 U/ml penicillin, and 100 μg/ml streptomycin in the presence or absence of rhTNF-α (0.01–100 ng/ml, R&D Systems, Abingdon, UK), or GM-CSF (10 ng/ml, R&D Systems, Abingdon, UK) as a positive control (37°C, 5% CO_2_). Dexamethasone (Glaxo-Wellcome-SKB, Stevenage, UK) (1 μM) was added to the eosinophil cultures together with TNF-α(50 ng/ml for degranulation and 20 ng/ml for survival and cytokines release) or with GM-CSF (10 ng/ml). After 22 hrs of culture in absence or presence of dexamethasone (1 μM) eosinophil survival was evaluated by Trypan blue exclusion test.

### Eosinophils degranulation and cytokines release

ECP level was measured in the culture supernatants by a RIA kit (ECP, Pharmacia Upjohn, Milton Keynes, UK). GM-CSF and IL-8 content was detected in the culture supernatants by ELISA kit (R&D Systems, Abingdon, UK).

### Adhesion assay

Eosinophils (10^4^/100 μl/well) were cultured in medium alone or with TNF-α (20 ng/ml) or GM-CSF (10 ng/ml) in the presence or absence of dexamethasone (1 μM) (30 min, 37°C) in 96 well plates pre-coated with recombinant VCAM-1, ICAM-1 or IgG. As a marker of adhesion EPO was detected as previously described [5]. Results are expressed as mean ± SEM.

### Statistical analysis

Statistical analysis was performed by Student's t paired test. A *p *value of <0.05 was considered statistically significant.

## Results

**Figure 1:**
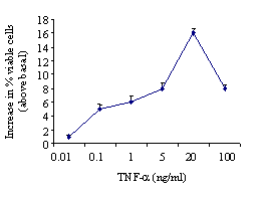
Effect of TNF-α on eosinophil viability *in vitro*. Eosinophils were incubated with different concentrations of TNF-α for 22 hrs. Eosinophil viability was evaluated by Trypan blue exclusion test. Values are expressed as percentage of survival increase in the presence of TNF-α *vs *medium alone. Values are mean ± SEM (n = 14).

TNF-α significantly increased eosinophil viability in a concentration-dependent fashion, compared to culture in medium alone, with a maximal effect at 20 ng/ml (Figure 1). This effect was not influenced by the addition of dexamethasone in the culture medium (13.3% *vs *11.5%).

**Figure 2:**
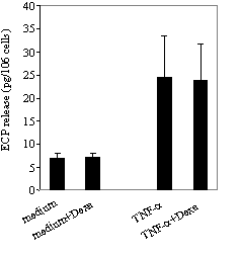
Effect of dexamethsone on TNF-α-induced ECP release from eosinophils. Eosinophils were cultured in medium alone (control) or with dexamethsone (medium+Dexa) or in the presence of TNF-α alone (50 ng/ml) (TNF-α) or TNF-α with dexamethsone (1 μM) (TNF-α+Dexa). ECP release was evaluated by RIA. Values are mean ± SEM (n = 3).

**Figure 3:**
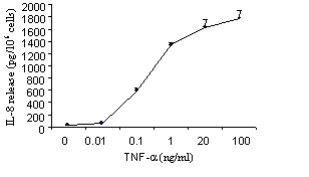
Effect of TNF-α on IL-8 release from eosinophils. Eosinophils were cultured with different concentrations of TNF-α (0 – 100 ng/ml). IL-8 release was evaluated by ELISA. Values are mean ± SEM (n = 5).

**Figure 4:**
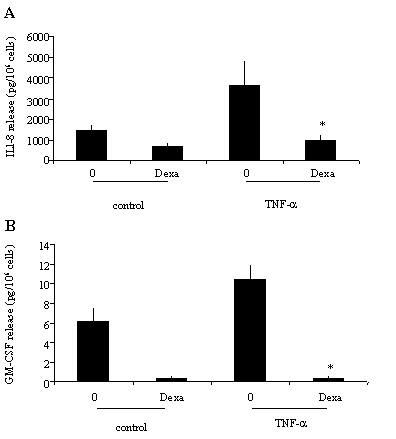
Effect of dexamethasone on TNF-α-induced IL-8 and GM-CSF release from eosinophils. Eosinophils were cultured with medium (control), or with TNF-α (20 ng/ml) (TNF-α) in the presence (Dexa) or absence (0) of dexamethasone (1 μM). IL-8 (**A**) and GM-CSF (**B**) release was evaluated by ELISA. Values are mean ± SEM (n = 5). *P < 0.05.

**Figure 5:**
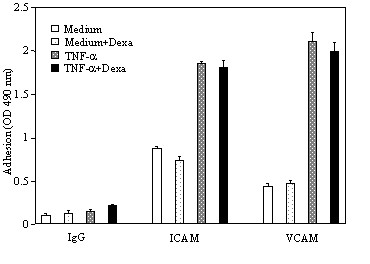
Effect of dexamethsone on TNF-α-induced eosinophil adhesion to VCAM-1 and ICAM-1. Eosinophils were cultured in medium alone or with dexamethsone, or with TNF-α (20 ng/ml) or TNF-α +dexamethsone in 96-wells plate coated with IgG, VCAM-1 and ICAM-1. EPO release was detected by colorimetric assay. Value are mean ± SEM (n = 3).

Incubation of eosinophils with TNF-α induced a significant release of ECP compared to eosinophils cultured in medium alone (24.5 ± 8.9 *vs *6.9 ± 1.2 pg/10^6^; *p *< 0.05). However, addition of dexamethasone in the cultures did not affect TNF-α-induced ECP release (Figure 2). The release of IL-8 by TNF-α treated eosinophils was dose-dependently proportional to the concentrations of TNF-α. A maximal release was achieved at 100 ng/ml (1770,49 ± 129 pg/ml; *p *< 0.05) (Figure 3). TNF-α also induced GM-CSF release by eosinophils although to a lesser extent than that of IL-8 (data not shown). Treatment of the cultures with dexamethasone completely blocked the TNF-α-induced release of both IL-8 and GM-CSF (*p *< 0.05) (Figures 4A–B). TNF-α enhanced significantly the percentage of eosinophil adhesion to VCAM-1, ICAM-1 and IgG in comparison to medium alone, by 177%, 205% and 169%, respectively. However, this effect was not inhibited by dexamethasone (Figure 5).

## Discussion

We have shown that dexamethasone inhibits the release of IL-8 and GM-CSF in TNF-α activated human peripheral blood eosinophils from non-atopic volunteers.

The roles of GM-CSF and IL-8 in allergic inflammation are well established. For example, GM-CSF is a potent survival factor for eosinophils and IL-8 is an important chemoattractant for neutrophils. The inhibitory effect of GCS on the production of GM-CSF, IL-8 and MCP-1 by eosinophils after different stimuli has been demonstrated [4]. However, its effect on eosinophils activated by TNF-α has not been fully investigated as yet. In our system we used dexamethasone to study the effect of GCS on TNF-α-induced eosinophil activation. It is important to note that different GCS have similar effects on inflammatory cells. Several reports have demonstrated that the inhibition of dexamethasone on eosinophil survival and activation parallels the one of inhaled GCS *in vitro*. For example, budesonide reduced the number of peripheral blood eosinophils by suppressing both their progenitors in the blood and colony-forming unit production in the bone marrow [6,7]. It is also known that inhalation of high doses of fluticasone reduced the number of blood eosinophils by increasing their apoptosis *in vivo *[8].

Although dexamethasone has been shown to induce eosinophil apoptosis, we have found that it did not decrease eosinophil survival after 18 h of treatment. Therefore, its effects on cytokine release observed in our system can not be attributed to the eosinophil death. Dexamethasone, as other GCS, inhibits cytokines release by eosinophils by interference with transcription factors such as NF-kB and AP-1 [9]. Since TNF-α is a potent inducer of NF-kB in eosinophils [10] we can hypothesize that dexamethasone inhibits GM-CSF and IL-8 release in TNF-α activated eosinophils by blocking NF-kB (genomic mechanism). In our study dexamethasone was unable to inhibit TNF-α-induced ECP release and their adhesion to immobilised VCAM-1, ICAM-1 and IgG. These data are in accordance with previous works in which dexamethasone did not affect the C5a- and IL-5 enhanced immunoglobulin-induced eosinophil release of EDN [11].

Recent evidence supports a direct and extremely rapid inhibitory effect of GCS on some activated inflammatory cells (i.e. basophils) via a non-genomic effect that results from the interaction of the GCS with biological membranes [12]. However, we have not observed any effect of dexamethasone on eosinophils degranulation and adhesion (rapid events).

In conclusion, from our data we can speculate that GCS exert beneficial effects in allergic inflammation also by selectively inhibiting TNF-α-induced eosinophils release of GM-CSF and IL-8, but not their survival, degranulation and adhesion.

## List of abbreviations used

TNF-α: tumor necrosis factor-α

GM-CSF: granulocyte-macrophage colony-stimulating factor

GCS: glucocorticosteroids

FCS: foetal calf serum

ECP: eosinophil cationic protein

VCAM-1: vascular cell adhesion molecule-1

ICAM-1: intercellular adhesion molecule-1

EDN: eosinophil-derived neurotoxin

## Competing interests

The author(s) declare that they have no competing interests.

## Authors' contributions

IU performed 80% of the experiments and organized the graphs. IP drafted the manuscript and organized the figures. VT performed 10% of the experiments. SJS contributed to the experiments and to draft the manuscript. DF performed the experiments of adherence. KPR participated in the design and coordination of the study. FLS performed 10% of the experiments, contributed to design and coordinate the study and to draft the manuscript.
